# The Psychology of Athletic Tapering in Sport: A Scoping Review

**DOI:** 10.1007/s40279-022-01798-6

**Published:** 2023-01-25

**Authors:** Maxwell J. Stone, Camilla J. Knight, Ross Hall, Catherine Shearer, Ross Nicholas, David A. Shearer

**Affiliations:** 1 School of Psychology, Newcastle University, Newcastle upon Tyne, UK; 2 Department of Sport and Exercise Sciences, Swansea University, Swansea, UK; 3 Welsh Institute of Performance Science, Sport Wales Institute, Cardiff, UK; 4 Faculty of Life Sciences and Education, University of South Wales, Pontypridd, UK; 5 Sport Wales Institute, Cardiff, UK; 6 Swim Wales, Swansea, UK; 7 Department of Sport Science and Physical Education, University of Agder, Kristiansand, Norway

## Abstract

Taper is a common training strategy used to reduce fatigue and enhance athletic performance. However, currently, no review has summarised what psychological research has been conducted examining taper, what this research shows and what future research needs to be undertaken to extend the field. Consequently, a scoping review was conducted with three aims: (a) to determine the characteristics of psychological research examining taper, (b) to summarise psychological research collected during taper with adult athletes and coaches, and (c) to identify gaps in psychological research examining taper. Forty-eight articles were identified following an exhaustive search strategy and charted following scoping review guidelines. Results showed most research was quantitative, used a longitudinal design, was conducted in swimming, triathlon, cycling or across multiple sports, and used a university-, regional- or national-level male athlete sample. Eight themes were developed to summarise the research: Mood, Perception of Effort, Perceived Fatigue and Wellness, Recovery-Stress, Taper as a Stressor, Stress Tolerance, Psychological Preparation and Cognitive Functioning. Additionally, four research recommendations were identified: (a) conducting exploratory research that examines the impact taper has on athletes’ and coaches’ competition preparation and stress experience, (b) asking more advanced psychological questions and conducting multi-disciplinary research, (c) including a more diverse participant sample in studies and (d) examining the impact of psychological interventions during taper. Overall, this scoping review has highlighted the limited research examining the psychology of taper and the need for focused research that asks more complex questions across diverse populations.

**Supplementary Information:**

The online version contains supplementary material available at 10.1007/s40279-022-01798-6.

## Key Points

**Table:** 

Most psychological research examining taper has been conducted from a physiological or training load monitoring perspective with male non-elite athletes.
Research suggests taper improves athletic functioning but may be a unique stressor for coaches. Both athletes and coaches psychological state during taper are likely influenced by personal and situational factors.
Key limitations associated with psychological research examining taper include a lack of constructs being investigated, findings not being critically discussed from a psychological perspective, only basic research questions being studied, lack of participant diversity and a lack of intervention-based research.

## Introduction

Taper refers to a progressive reduction in training load prior to athletic competition aiming to reduce fatigue while maintaining/enhancing training adaptations [1]. This reduction in fatigue and maintenance/enhancement of training adaptions has a substantial impact on athletic performance, normally leading to improvements of 2–3% [1]. Although these changes are marginal, in elite sport, they often differentiate between finishing inside or outside medal positions during major sports events [2]. Consequently, understanding and optimising taper is key to athletic success and has subsequently received considerable research attention [3, 4].

Research examining taper has predominantly focused on either identifying the optimal taper strategy or identifying variables underpinning taper’s performance-enhancing effects [3, 4]. For example, a meta-analysis aiming to find the optimal taper found that a 8–14 day progressive reduction in training volume of 41–60%, which maintains training intensity and frequency, was best for most swimmers, runners and cyclists [3]. Additionally, a host of physiological variables have been studied during taper, including cardiorespiratory, metabolic, biochemical, hormonal, neuromuscular and immunological factors [4]. Of these variables, taper appears to be consistently associated with improvements in blood creatine kinase, testosterone, cortisol, and muscular strength and power, suggesting these physiological variables underpin performance improvements during taper [5–7].

Alongside physiological variables, psychological variables have also been proposed as key to understanding taper [4]. Indeed, researchers have examined mood, perception of effort, recovery-stress and sleep quality, with findings from this research typically showing these variables improve following taper [8–10]. For example, one study found mood and performance to significantly improve after 14 days of taper with eight cyclists [8]. In another study, 16 triathletes reported significant improvements in total stress and total recovery alongside increased 3-km time trial run performance after a 2-week taper [9]. Finally, a 1-week taper led to significant improvements in the heart rate:perceived exertion ratio and performance of collegiate cyclists [10]. Cumulatively, this research suggests athletes’ psychological state improves during taper and may underpin taper’s performance-enhancing effects. Developing a better understanding of the psychological changes associated with tapering, including how they relate to known physiological effects, which have received more research attention, is key to ensuring it is optimally implemented and performance maximised.

Despite an understanding of the psychology of taper being important, currently, little knowledge is available detailing what types of psychological research has been conducted, what this research demonstrates and what further research needs to be done. For instance, the last review to be conducted summarising psychological research associated with taper [4] was not systematic, did not analyse the characteristics of psychological research and did not suggest future research areas. Consequently, it is unknown whether all relevant research was identified (and how it was identified), whether there are re-occurring methodological issues associated with psychological research examining taper and where researchers need to focus their attention to further the field. Furthermore, and compounding these three issues, the review is over a decade old and consequently may offer an outdated summary of psychological research examining taper.

Overall, there is a lack of a systematic up-to-date review that summarises psychological research examining taper and identifies future research needs. This represents a significant gap in knowledge as it makes it difficult for researchers to optimally study the psychology of taper, which in turn prevents taper from being effectively implemented. As such, a scoping review was conducted with three aims. First, to determine the characteristics (e.g. designs, participants) of psychological research examining taper. Second, to summarise psychological research examining taper conducted with adult athletes and coaches. Third, to identify gaps in psychological research examining taper and suggest areas of future research.

## Methods

We defined a scoping review as a review addressing a broad research question that maps key concepts, types of evidence and gaps in research via systematic searching, selecting and synthesising of knowledge [11]. A scoping review was considered appropriate as its purpose was consistent with our aims of mapping the volume and characteristics of a research area, summarising research relating to a topic and identifying gaps in knowledge [12, 13].

The method for this scoping review was informed by the Arksey and O’Malley [13] framework and consisted of five phases: (a) identifying the research question, (b) identifying relevant studies, (c) study selection, (d) charting the data and (e) collating, summarising and reporting the results. For an overview of the review protocol, please see the Electronic Supplementary Material (ESM). The review was conducted in accordance with recent scoping review reporting guidelines (PRISMA-ScR; [14]). All items of the reporting guidelines were met (see ESM), excluding items relating to critical appraisal (items 12 and 16) that are considered optional in scoping reviews [14]. This review was prospectively registered with the Open Science Framework on 17 March, 2021 (https://osf.io/xchpg).

### Identifying the Research Question

This review was guided by the questions: (a) what types of psychological research examining taper has been conducted in adult sport, with whom and with what measures? (b) what has research examining the psychology of taper found? and (c) where are the gaps in knowledge and how can they be addressed? These questions were developed to clarify the concept (taper), target population (adult sport participants) and outcomes (research findings and future directions) [15], as well as to ensure the limitations associated with the existing understanding of the psychology of taper were addressed.

### Identifying Relevant Studies

To identify sources relevant to the research question, articles were identified via five sources: (a) electronic databases (Scopus, 1788–present; Web of Science, 1900-present; PsycArticles, 1985–present; PsycInfo, 1806–present; SportDiscus, 1892–present; and PubMed, 1975–present), (b) manual searching of journals (*Psychology of Sport and Exercise, Sport, Exercise, and Performance Psychology, Journal of Sport and Exercise Psychology, Journal of Applied Sport Psychology, International Journal of Sport and Exercise Psychology, Journal of Clinical Sport Psychology, Journal of Sport Psychology in Action*), (c) reference list inspections, (d) “cited by” searches and (e) from previous knowledge. The six electronic databases were chosen to provide coverage of sport science, psychology and multidisciplinary publications relevant to the research question and identified by the research team in consultation with a university librarian with specialist knowledge of sports science and psychology databases.

Searches of electronic databases were first performed on 27 March, 2021 and then again on 5 November, 2021. Database abstracts, keywords and titles were searched using a combination of taper*, psych*, mental*, mental skills, cogniti*, emoti*, behav*, sport*, recover*, fatigu*, prepar*, peak*, optim*, pre-perform*, compet*, train*, athlete* and coach*. In databases that allowed for it (SportDiscus and PsycInfo), exploding subject heading searches were performed. See Table 1 for the complete search terms used for PsycInfo.

**Table 1:** Complete search terms used for APA PsycInfo

Search ID#	Search term
1	"taper*".ab,id,ti
2	"psych*".ab,id,ti
3	"mental*".ab,id,ti
4	mental skills.ab,id,ti
5	"cogniti*".ab,id,ti
6	"emoti*".ab,id,ti
7	"behav*".ab,id,ti
8	"sport*".ab,id,ti
9	"pre-perform*".ab,id,ti
10	"recover*".ab,id,ti
11	"fatigu*".ab,id,ti
12	"compet*".ab,id,ti
13	"train*".ab,id,ti
14	"athlet*".ab,id,ti
15	"coach*".ab,id,ti
16	exp Cognition/
17	exp Emotions/
18	exp Behavior/
19	exp Sports/
20	exp Fatigue/
21	exp Competition/
22	exp Athletic Training/ or exp Training/
23	exp Sport Psychology/ or exp Psychology/
24	exp College Athletes/ or exp Athletes/ or exp Professional Athletes/
25	exp Coaches/
26	2 or 3 or 4 or 5 or 6 or 7 or 16 or 17 or 18 or 23
27	8 or 9 or 10 or 11 or 12 or 13 or 14 or 15 or 19 or 20 or 21 or 22 or 24 or 25
28	1 and 26 and 27

*ab* abstract, *exp* exploding subject headings,* id* key concepts, *ti* title

Asterisk indicate Boolean searching

Manual searches of journals were first conducted on 29 March, 2021 and then again on 5 November, 2021 using the online search function on journal websites and using the search term “taper”. Manual searching involved all aspects of a journal’s database (e.g. title, abstract, keyword, full text). A broad and simplistic search term was used to identify the maximum number of relevant articles. Complete search terms, date coverage and search limits used for each electronic and journal database can be found in the ESM.

The reference lists of ten fully screened articles (i.e. those that had passed title, abstract and full-text screening) were manually searched for additional articles [16–25]. These articles were specifically chosen to ensure an even spread of publication dates across the whole publication period, therefore maximising the likelihood of finding missing articles. “Cited by” searches were also conducted on these ten articles via Google Scholar with the first 100 records being screened for appropriateness in relation to the research questions. Like other scoping reviews [26], limited (i.e. only ten articles searched and only 100 records screened) reference list searches and “cited by” searches were conducted because we felt the comprehensive nature of the database screening meant it was unlikely that further searches would return relevant articles. Additionally, the latest review summarising psychological factors associated with taper was also manually checked for potentially relevant articles [4]. Other potentially relevant articles were identified using the existing knowledge of the research team.

### Study Selection

Consistent with recommendations, a two-stage screening process was used in this scoping review to select studies [13]: title and abstract, and full text. During each stage, to be eligible in this scoping review, articles needed to (a) be written in English, (b) be peer-reviewed original articles (including in press articles), (c) examine (quantitatively or qualitatively) psychological factors (i.e. cognitive, emotional, or behavioral factors), (d) be conducted during taper; [1]) or have findings referencing taper, (e) have participants over the age of 18 years and (f) have participants who are athletes (i.e. individuals competing in sport) or sport coaches (i.e. individuals involved in the training of athletes in sport) involved in sport (i.e. competitive physical activity requiring skill and/or physical prowess). Only articles written in English were included as we did not have translation services available. Similarly, only peer-reviewed original articles were included as we were only interested in research that had passed scientific scrutiny. An adult-only sample was chosen as developmental and adult athletes differ physiologically and psychologically [27], and therefore pooling data may confuse understanding. Research that was conducted during taper or referenced taper was used to capture all relevant research related to the studies aims. Psychological research and research that had either an athlete or coaching sample was chosen to ensure only articles relevant to the research questions were identified. No restriction was placed on sport type, study design, methodology (e.g. cross-sectional or longitudinal) or publication date.

Identified articles were imported into the web-based systematic review manager Covidence (https://www.covidence.org/) for title, abstract and full-text screening. Article titles and abstracts were screened by two reviewers for appropriateness using the eligibility criteria. For articles where there was disagreement, the disagreeing reviewers used the notes function in Covidence explaining their decisions. The reviewers then examined these notes, and a decision was reached whether the article should be included. If no agreement could be made, a third reviewer screened the article, reviewed the notes, and performed a concluding decision. Articles considered relevant after title and abstract screening progressed to a full-text review. Articles that could not be accessed were requested from the original authors. After passing a full-text review, study characteristics were charted.

### Charting the Data

Data were charted by the lead author using Microsoft Excel (ESM) and reviewed by the research team. The data chart consisted of relevant study characteristics to be extracted from articles, including author(s), publication date, journal title, study aim, study design, study methodology, number of participants, number of male participants, number of female participants, participant descriptor, sport, taper duration, psychological measure/data collection method, psychological findings, conclusion, strengths and limitations. Full-text screening and data charting were performed by the lead author; however, discussions took place when questions arose about the data charting process.

### Collating, Summarising and Reporting the Results

Consistent with scoping review recommendations, we conducted a frequency analysis to identify the characteristics of psychological research examining taper and content analysed article findings to aid with the collation, summarising and reporting of results [13, 15]. Specifically, we used a conventional content analysis in which we examined the findings of the identified articles and organised them into re-occurring concepts and ideas [28, 29]. A conventional content analysis was chosen because it is recommended when trying to describe a phenomenon, therefore matching the aims of this scoping review [28]. Organisation of re-occurring concepts and ideas was done both deductively, drawing upon previous understanding [9], and inductively, when existing knowledge could not account for the novelty of the data. Where appropriate, athlete and coach data were synthesised to provide a greater understanding. Findings were reviewed by the research team to ensure theme titles and their content were conceptually consistent.

## Results

### Search and Selection of Articles

**Fig. 1:**
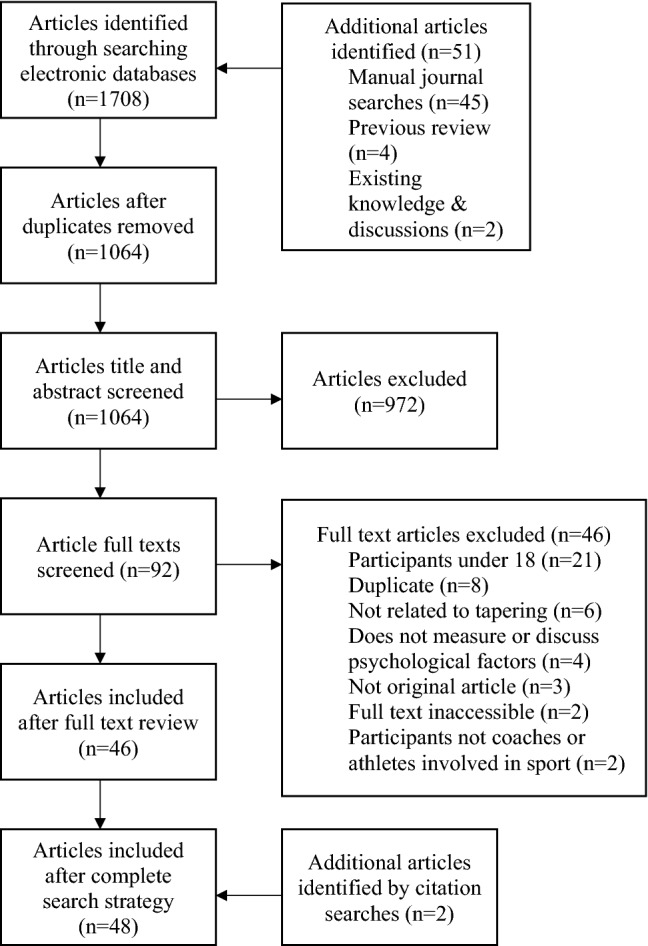
Flowchart of search process

The electronic database searches returned 1708 results and additional article searches (excluding citation based searches) returned 51 results. Of these 51 additional articles, 45 were identified via manual journal searches, four via a previous review [23, 30–32] and two via existing knowledge and discussions with the research team [22, 25]. After deduplication in Covidence, 695 articles were removed, leaving 1064 articles for title and abstract screening. Following title and abstract screening, 972 articles were excluded from the review because of not meeting the eligibility criteria, resulting in 92 articles being left for full-text screening. Most articles were removed at title and abstract screening because of the search query returning unrelated results in disciplines such as engineering and drug efficacy. Full texts for two articles could not be retrieved and were therefore excluded from full-text screening. After full-text screening, 46 articles were excluded resulting in 46 articles being retained for data charting. Two additional articles were identified via “cited by” searches [33, 34], resulting in a total of 48 articles being identified and charted (See Fig. 1 for details of the identification and screening progress).

### Characteristics of Psychological Research Examining Taper

Included articles were published between 1989 and 2020, with 75% being published after 2005. Most (52%) articles were published in *Medicine and Science in Sports and Exercise* (*n* = 6), *Journal of Strength and Conditioning Research* (*n* = 6), *International Journal of Sports Medicine* (*n* = 5), *Journal of Sports Sciences* (*n* = 4) and *Journal of Applied Sport Psychology* (*n* = 4). Most (79%) articles collected quantitative data (*n* = 38); however, qualitative (*n* = 7) and mixed method (*n* = 3) were also collected.

Of the quantitative research conducted, most (95%) used a longitudinal design (*n* = 36), with the remaining studies using a cross-sectional design (*n* = 2). Longitudinal quantitative research consisted of experimental (*n* = 20), field-based (*n* = 12) and quasi-experimental (*n* = 2) research. Of the experimental research conducted, both with (*n* = 11) and without (*n* = 9) control group methodologies were reported. Of the qualitative research conducted, most (71%) did not report or make explicit the specific methodological (e.g. grounded theory) or philosophical framework (e.g. interpretivism) used. Two qualitative studies did report their philosophical approach, using interpretive constructionism and pragmatism, respectively.

Most (92%) articles used an athlete sample (*n* = 44), with only three articles examining coaches and one article examining both athletes and coaches. In total, the articles included 1588 participants. Of these participants, 1548 were athletes (97%) and 40 were coaches (3%). Of the athletes, 1026 were male (66%) and 475 were female (31%). Of the coaches, 28 were male (70%) and 6 were female (15%). Across four studies [17, 35–37], the gender of 47 athletes and six coaches was not reported or made explicit (representing 3% and 15% of the respective total percentages).

To report participant descriptors and sports more coherently, participant descriptors and sports that shared similar semantic meanings were combined (e.g. participants described as “Elite”, “Olympic and Paralympic” and “World Class” were categorised together as “Elite” and “Rugby League”, “Rugby Sevens” and “Rugby Union” were categorised as “Rugby”). Where participants were given a dual description by the original authors (e.g. regional-national), the lowest descriptor was used. Articles that used athletes from multiple sports were categorised as “multi-sport”. Using this classification system, the most (27%) reported participant descriptor was elite (*n* = 13) [38]. However, overall, most (52%) psychological research used a non-elite sample consisting of university- (*n* = 12), regional- (*n* = 7) or national-level (*n* = 6) athletes. The most reported sport of participants were swimming (*n* = 11), multi-sport (*n* = 7) and triathlon (*n* = 6). One article did not report or make explicit the sport of participants to protect their anonymity [22].

Taper durations ranged from 2 [39] to 70 [40] days. However, the most reported taper durations were 7 (*n* = 10), 14 (*n* = 8) and 21 (*n* = 6) days. Two articles reported multiple tapers with varying lengths [41, 42]. Five articles did not report or make explicit the duration of taper [24, 25, 31, 43, 44]. A summary of the characteristics of psychological research examining taper can be found in Table 2.

**Table 2:** Study characteristics of psychologically related taper research

Study characteristic	*n*	%
**Publication year**		
1989–1994	6	13
1995–2000	4	8
2001–2006	4	8
2007–2012	9	19
2013–2018	17	35
2019–present	8	17
**Journal**		
Journal of Strength and Conditioning Research	6	13
Medicine and Science in Sports and Exercise	6	13
International Journal of Sports Medicine	5	10
Journal of Applied Sport Psychology	4	8
Journal of Sports Sciences	4	8
Scandinavian Journal of Medicine and Science in Sports	3	6
Biology of Sport	2	4
International Journal of Sports Science and Coaching	2	4
Journal of Science and Medicine in Sport	2	4
Applied Physiology, Nutrition, and Metabolism	1	2
Canadian Journal of Applied Physiology	1	2
European Journal of Sport Science	1	2
International Journal of Sport and Exercise Psychology	1	2
International Journal of Sports Physiology and Performance	1	2
International Journal of Wrestling Science	1	2
Journal of Human Kinetics	1	2
Journal of Sports Medicine and Physical Fitness	1	1
Journal of the American College of Nutrition	1	2
Perceptual and Motor Skills	1	2
Psychology of Sport and Exercise	1	2
Psychoneuroendocrinology	1	2
Sports Medicine - Open	1	2
The Sport Psychologist	1	2
**Design**		
Quantitative	38	79
Qualitative	7	15
Mixed method	3	6
**Methodology**		
Longitudinal experiment with control group	11	23
Longitudinal field study	11	23
Longitudinal experiment without control group	9	19
Not reported/explicit	5	10
Case study	2	4
Longitudinal quasi-experiment	2	4
Survey	2	4
Cross-sectional field study	1	2
Cross-sectional quasi-experiment	1	2
Interpretive constructionist	1	2
Longitudinal field study with experimental (no control group) follow-up	1	2
Longitudinal field study with follow-up focus groups	1	2
Pragmatism	1	2
**Participant**		
Athlete	44	92
Coach	3	6
Athlete and coach	1	2
**Participant** **gender**		
Male	1062	67
Female	496	31
Not reported/explicit	30	2
**Participant descriptor**		
Elite	13	27
University	12	25
Regional	7	15
National	6	13
Professional/competitive	3	6
Trained	3	6
High level	2	4
International	2	4
**Participant sport**		
Swimming	11	23
Multisport	7	15
Triathlon	6	13
Cycling	5	10
Rugby	3	6
Running	2	4
Australian Rules	1	2
Canoeing	1	2
CrossfFit	1	2
Endurance sport	1	2
Mountain biking	1	2
Not reported/explicit	1	2
Rowing	1	2
Soccer	1	2
Strongman	1	2
Tennis	1	2
Track and field	1	2
Water polo	1	2
Weightlifting	1	2
Wrestling	1	2
**Taper duration (days)**		
2	1	2
7	10	21
10	2	4
14	8	17
21	6	13
28	4	8
70	1	2
n/a	9	19
Not reported/explicit	5	10
Multiple	2	4
**Psychological measure/data collection method**		
***Mood***		
Profile of Mood State	18	26
Brunel Mood Scale	2	3
***Perceived exertion***		
Rating of Perceived Exertion	10	14
Action Crisis Scale	1	1
Feeling Scale	1	1
Felt Arousal Scale	1	1
Form Scale	1	1
Short Flow State Scale	1	1
***Perceived wellness and fatigue***		
Fatigue, sleep quality, muscle soreness, stress levels and mood	2	3
Perceived well-being in the legs	2	3
Training, sleep, leg pain, infection, concentration, efficacy, anxiety, irritability and general stress	2	3
Mood state, energy levels, stress, fatigue and muscle soreness	1	1
Pain, recovery and fatigue	1	1
Sleep quality, readiness to train, general muscular soreness, fatigue, stress, mood and motivation	1	1
***Recovery-stress***		
Recovery-Stress Questionnaire-Sport (72 item)	6	9
Recovery-Stress Questionnaire-Sport (52 item)	1	1
Short Recovery and Stress Questionnaire	1	1
***Stress tolerance***		
Daily Analysis of Life Demands	4	6
***Cognitive functioning***		
STROOP task	1	1
***Qualitative***		
Semi-structured interviews	7	10
Focus groups	1	1
***Survey***		
Open and closed questions asking about tapering practices	2	3
***Other***		
Athlete Burnout Questionnaire	1	1
State-Trait Anxiety Inventory	1	1

Note: Due to rounding some total percentages do not equate to 100%

### Summary of Psychological Research Examining Taper

In total, eight themes were developed via content analysis [29]: Mood, Perception of Effort, Perceived Fatigue and Wellness, Recovery-Stress, Taper as a Stressor, Stress Tolerance, Psychological Preparation and Cognitive Functioning. Themes are presented in order of the volume of research underpinning them, with larger themes first. See Table 3 for a summary of independent articles’ main findings.

**Table 3:** Main findings from psychologically related taper research

Authors (date)	Methodology and participants	Measure/data collection method	Main findings
Houmard et al. [17]	Quantitative, longitudinal field study with experimental (no control group) follow up with 5 university cross-country runners	RPE	No significant difference in RPE between midseason, post-championship and reduced training
O’Connor et al. [45]	Quantitative, longitudinal quasi-experiment with two groups (normal training and control) with 22 college and university swimmers	POMS	Mood improved after taper. No change in mood of controls. Tension remained elevated above baseline after taper. Cortisol and depression unrelated during taper
Raglin et al. [25]	Quantitative, longitudinal quasi-experiment with three groups (dropouts, unsuccessful adherers, and successful adherers) with 84 freshmen rowers	POMS	Significant effect of time on total mood, tension, vigour and fatigue. No significant difference in mood between groups (adherers vs non-adheres). Mood of unsuccessful adherers was significantly elevated above baseline after taper
Raglin et al. [31]	Quantitative, cross-sectional (multiple samples across different time points) field study with 186 university swimmers	POMS	Each mood factor improved after taper. Tension remained elevated throughout the seasons. Gender significantly influenced mood responses across several seasons. However, when data across seasons were pooled, no gender effect found. Tension significantly higher in women after taper across all seasons
Berglund and Safstrom [44]	Quantitative, longitudinal field study with 14 world class canoeists	POMS and questionnaire measuring perceptions of POMS as a training titration tool	Significantly improved mood after taper. Initially doubtful of use of POMS but then became positive. Thought the use of the POMS to titrate training load had a positive impact on performance
Flynn et al. [46]	Quantitative, longitudinal field study with 13 collegiate cross-country runners and swimmers	POMS and RPE	Unchanged mood and RPE after taper for runners. Significantly improved mood and RPE after taper for swimmers
Raglin et al. [43]	Quantitative, longitudinal field study with 12 university swimmers	POMS	Mood returned to baseline after taper. Mood was significantly negatively correlated with anaerobic swimming power throughout the study duration
Taylor et al. [24]	Quantitative, longitudinal field study with 7 national swimmers	POMS	Tension, depression, confusion and vigour significantly worse after taper compared with peak training
Martin and Andersen [10]	Quantitative, longitudinal experiment without control group with 11 college and category 3 licenced cyclists	RPE	RPE-power intercept significantly increased, and the magnitude of the slope significantly decreased after taper. RPE-HR intercept significantly increased, but there was no change in slope, after taper
Martin et al. [47]	Quantitative, longitudinal experiment without control group with 11 college and category 3 licenced cyclists	POMS	No significant change in mood
Margaritis et al. [30]	Quantitative, longitudinal experiment with a control group (two groups: placebo/control and supplementation) with 24 competitive triathletes	POMS	Mood significantly improved after taper
Neary et al. [23]	Quantitative, longitudinal experiment without a control group (three groups: 30%, 50% and 80% reduction in training load) with 11 competitive cyclists	RPE	No significant difference in RPE across condition or time. RPE-HR significantly improved in the 50% reduction in training load group at post taper, compared with pre-taper
Hanton et al. [22]	Qualitative (methodology not reported/explicit) with 10 international athletes	Semi-structured interviews	Athletes thought it was important to have a good start at competition and this told them their taper was right. Competitive stressors identified by athletes included poor mental, physical and technical preparation
Cresswell and Eklund [35]	Qualitative (methodology not reported/explicit) 15 professional rugby union players	Semi-structured interviews	Transition from taper to base fitness work was identified as a source of burnout
Atlaoui et al. [48]	Quantitative, longitudinal field study with 13 national or international swimmers	Perceived fatigue and wellness questionnaire (perceptions of training, sleep, leg pain, infection, concentration, efficacy, anxiety, irritability and general stress)	Changes in total score of fatigue between intense training and taper was negatively related to high frequency heart-rate variability and positively related to low frequency heart-rate variability and the LF/HF ratio
Coutts et al. [49]	Quantitative, longitudinal experiment with a control group (two groups: normal training/control and intense training) with 16 national or international triathletes	DALDA	No significant group or time difference in sources of stress. Intense training group reported significant improvement in symptoms of stress after taper
Coutts et al. [9]	Quantitative, longitudinal experiment with a control group (two groups: normal training/control and intense training) with 16 national or international triathletes	RESTQ-Sport-76	Intense training group reported significantly improved stress, lack of energy, physical complaints, fitness/injury, fitness/being in shape after taper, compared with the control group
Coutts and Raeburn [50]	Quantitative, longitudinal experiment with a control group (two groups: normal training/control and intense training) with 20 state rugby league players	RESTQ-Sport-76	Intense training group reported significantly improved physical recovery, general well-being, fatigue, and disturbed breaks after taper compared with pre-taper
Olusoga et al. [51]	Qualitative (methodology not reported/explicit) with 12 world class diving, sailing, swimming, bowls, equestrian, field hockey, lacrosse or table tennis coaches	Semi-structured interviews	Managing athletes psychologically and preparing athletes for competition were stressors identified by coaches. One coach noted that “making sure the taper is right” was a key concern
Santhiago et al. [20]	Quantitative, longitudinal field study with 10 elite swimmers	POMS	Vigour was significantly worse after taper compared with pre-taper
Zehsaz et al. [52]	Quantitative, longitudinal experiment with a control group (two groups: control and three weeks of taper) with 24 elite cyclists	POMS	Mood significantly improved in the taper group. Mood was significantly better in the taper group after weeks 1 and 2 of taper, compared with the control group
Dupuy et al. [53]	Quantitative, longitudinal experiment without a control group with 11 provincial endurance sport athletes	STROOP, POMS and RESTQ-Sport-76	Fatigue, vigour and energy index returned to baseline after taper. Reaction time significantly improved after taper. RPE remained unchanged
Tobar [54]	Quantitative, cross-sectional (10 years) quasi experiment (two groups: high and low trait anxiety) with 159 university swimmers	POMS and STAI	Depression, anger and total mood significantly improved after taper for both groups. Low trait anxiety swimmers reported significantly less anger after taper compared with baseline. High trait anxiety swimmers reported significantly less depression after taper compared with baseline. Tension remained elevated after taper in both groups. Female swimmers reported less fatigue than men after taper, compared with baseline. Female swimmers reported more tension after taper compared with male swimmers. Compared with low trait anxiety swimmers, high trait anxiety swimmers reported less vigour and more confusion
Dupuy et al. [55]	Quantitative, longitudinal experiment without control group with 11 provincial endurance sport athletes	POMS, RESTQ-Sport-76 and RPE	RPE remained unchanged. Vigour, energy index and fatigue significantly improved after taper. General stress, sport-specific stress, general recovery and sport-specific recovery significant improved after taper
Gomes et al. [36]	Quantitative, longitudinal experiment without control group with 10 national tennis players	DALDA	Symptoms of stress significantly improved after taper
Karimi et al. [34]	Quantitative, longitudinal experiment with a control group (three groups: control, 50% taper, and 75% taper) with 30 high-level wrestlers	BRUMS	Significant group or time effect on mood (specific findings unclear)
Kennedy et al. [41]	Mixed-method, longitudinal field study with follow up focus groups with 25 university swimmers	Form scale and focus groups	Perceived form significantly improved after taper 2 compared with taper 1, build 2 and build 3. Perceived feeling significantly improved after taper 2 compared with build 1, taper 1, build 3 and taper 3. Perceived feeling significantly improved after taper 3 compared with build 1 and taper 1. Energy level significantly improved after taper 2 compared with any other period
Anderson et al. [37]	Qualitative (methodology not reported/explicit) with 17 elite athletes and 6 elite coaches in rowing, swimming or diving	Semi-structured interviews	One athlete reported identifying their optimal psychological state through taper. Optimal psychological state prior to competition was characterised by feeling confident, having good body language, the right level of nerves, feeling able to cope and a sense of calm. Physical and psychological preparation, training base and psychological recovery were factors identified as contributing to a peak performance. Poor preparation and a lack of recovery were identified as factors preventing an optimal psychological state. Being in the correct psychological state prior to performance was considered key to optimal psychological states during competition
Aubry et al. [19]	Quantitative, longitudinal experiment with a control group (two groups: control and intense training) with 40 regional or national triathletes	POMS and RPE	Unclear changes in RPE. Fatigue and energy index of acutely fatigued athletes improved, returning to baseline values, after taper. Unclear within- and between-group differences in vigour
Hausswirth et al. [56]	Quantitative, longitudinal experiment with a control group (two groups: control and overload training) with 27 well-trained triathletes	POMS	Athletes diagnosed as functionally overreached reported significantly less fatigue compared with pre-taper (end of overload)
Crowcroft et al. [57]	Quantitative, longitudinal experiment with a control group (two groups: control and heat and hypoxic) with 18 well-trained triathletes	RPE and perceived fatigue and wellness questionnaire (pain, recovery and fatigue)	Small likely greater improvement in pain in the control group compared with the heat and hypoxic group. No clear differences between groups in recovery, fatigue or RPE
Cruickshank and Collins [58]	Qualitative, pragmatism with 15 elite rugby union, rugby league, soccer, and Olympic individual and team sport managers, head coaches or performance directors	Semi-structured interviews	Optimal use of dark side behaviour was determined by contextual and interpersonal awareness of when to use them. One performance director said they would not use dark side behaviours during taper as they had to “get on and deal with what’s there.”
Bellenger et al. [59]	Quantitative, longitudinal experiment without a control group with 15 local runners and triathletes	DALDA and perceived fatigue and wellness questionnaire (mood state, energy levels, stress fatigue and muscle soreness)	Fatigue, muscle soreness, worse-than-normal symptoms of stress and energy levels almost certainly improved after taper. Possible or very likely trivial changes in mood and stress after taper
Bouaziz et al. [60]	Quantitative, longitudinal field study with 16 elite Rugby Sevens players	Perceived fatigue and wellness questionnaire (perceptions of training, sleep, leg pain, infection, concentration, efficacy, anxiety, irritability and general stress)	Total score of fatigue significantly improved after taper and positively correlated with the cortisol: cortisone ratio
Flatt et al. [61]	Quantitative, longitudinal field study with 10 collegiate swimmers	Perceived fatigue and wellness questionnaire (perceived sleep quality, fatigue, muscle soreness, stress and mood)	Sleep, fatigue and muscle soreness significantly improved after taper compared with overload measurements. Sleep significantly improved after taper compared with baseline measurements
Myers et al. [62]	Quantitative, longitudinal field study with 10 international swimmers	BRUMS and RESTQ-Sport-76	Total mood disturbance lowest after taper. Slope for vigour and fatigue was increasing and decreasing, respectively, over time. Improvement in total stress and total recovery after taper. Slope for fatigue, emotional exhaustion and injury decreased over time. Slope for social relaxation and personal accomplishment increased over time. BRUMS fatigue, fitness, injury and fatigue (RESTQ-Sport-76) had a significant negative relationship with FINA points over time
Ritchie et al. [18]	Qualitative (methodology not reported/explicit) with 7 Olympic and Paralympic track and field coaches	Semi-structured interviews	Psychological preparation was integrated into taper planning. Confidence considered important during taper, with one coach noting “a lot of tapering is about confidence.” Mentally preparing athletes for competition was a conscious decision during taper. One coach noted that “we have to get athletes where they are empowered and mentally strong.” Coaches reported using positive feedback and giving information about training they had done to increase confidence. Coaches also reported using specific confidence building sessions during taper. Coaches monitored athletes psychological state, such as their confidence, throughout taper. Taper was considered a collaborative process, and the quality of the coach athlete relationship was considered key for effective tapering. Specifically, honesty, appropriate praise and being there for the athlete were highlighted as important. Coaches also thought it was important athletes were confident in their coaches. Psychological challenges during taper included maintaining an optimal psychological state, managing over and under confidence, “dealing with boredom” and maintaining a focus on preparation. Coaches also thought their mental state could be a challenge. For example, not being negatively affected by the environment and maintaining composure and control around athletes were identified as specific challenges
Rønnestad et al. [63]	Quantitative, case study with 1 elite cross-country mountain biker	Perceived fatigue and wellness questionnaire (well-being of the legs), RPE	Perceived feeling in legs progressively got worse during the overload and then progressively improved during taper. RPE improved during taper
Venhorst et al. [39]	Quantitative, longitudinal experiment with a control group (two groups: drop task and control/taper) with 11 local runners	Perceived fatigue and wellness questionnaire, FS, FAS, ACRISS, FSS	Compared with the drop task group, the control group experienced significantly better muscle discomfort, unpleasantness, perceived physical strain, perceived mental strain and positive valence following the 20-km time trial
Winwood et al. [64]	Mixed-method, open and closed survey questions with 454 regional-professional strongman athletes	Tapering practices of strongman athletes survey	Psychological readiness (feeling rested and mentally prepared) highlighted as a reason for tapering. For example, one athlete said, “To create the ‘itch/desire’ for competition” and another said, “come into the contest mentally and physically ready”
Botonis et al. [33]	Quantitative, longitudinal field study with 8 high-level water polo players	Perceived fatigue and wellness questionnaire (fatigue, sleep quality, muscle soreness, stress levels and mood)	Wellness significantly improved after taper compared with the overload phase. Wellness across both weeks of taper stayed relatively unchanged. Throughout the training period, daily internal training load was moderately negatively correlated with morning wellness scores. Significant positive correlations between % reduction in internal training load between the overload and taper period and wellness scores. Same finding, albeit a negative relationship, found for sleep quality
Figueiredo et al. [65]	Quantitative, longitudinal field study with 16 elite soccer players	DALDA (only part B)	Almost certain improvement in stress tolerance after taper compared with the overload period. Very likely improvement in stress tolerance after taper compared with the baseline period. Non-significant correlation between lnRMSSD and stress tolerance during taper
Rønnestad and Vikmoen [42]	Quantitative, longitudinal experiment with a control group (two groups: 6-day overload + 5-day taper or 11-day taper/control) with 17 elite cyclists	POMS, perceived fatigue and wellness questionnaire (well-being of the legs), RPE	No significant changes in RPE. Fatigue significantly improved after taper compared with pre-taper, in the experimental group. No other significant changes in mood after taper in either group. No significant within-group changes in perceived well-being in legs. At day 11, experimental group had better perceived well-being in legs compared with the control group. Improvement in well-being of legs after taper was greater in the experimental group compared with the control group
Travis et al. [66]	2 national weightlifters	SRSS	Both athletes reported higher improved mean recovery and stress scores after taper compared with baseline measurements
Wilson et al. [67]	Qualitative, interpretive constructionist with 7 elite half pipe snowboarders, swimmers, ice skaters, downhill mountain bikers, trampolinists or rock climbers	Semi-structured interviews	Athletes highlighted preparing for competition as requiring mental toughness as it involves focusing on the present moment to avoid distractions. Athletes reported using self-compassion during taper to cope with difficulties through reflection, acceptance, understanding, self-care, personal detachment and re-appraisal. One athlete reported using self-compassion during taper to manage self-criticisms, prevent rumination, and enhance self-belief. The athlete said “It’s important to be mentally tough, like hard training and getting through hard workouts. I can be a little bit hard on myself during the taper time where you expect everything to go smoothly. At that point you have to use self-compassion. You’ve already done the training and you have been mentally tough, but then you have to change your mind so you trust what you have done and believe that it has worked and you bring down the volume and start to feel better … I’m not saying that during hard training you don’t have self- compassion or even within the taper time you don’t need to be mentally tough. But I think in the timing, sometimes I’ll just in- instinctively use one more than the other.”
Campbell et al. [16]	Quantitative, longitudinal experiment without a control group with 13 trained Australian Rules players	Perceived fatigue and wellness questionnaire (sleep quality, readiness to train, general muscular soreness, fatigue, stress, mood and motivation), POMS, RESTQ-Sport-52	Wellness significantly worse after taper compared with normal training. Moderate effect for recovery between intense training and tapered training. Readiness to train, total mood disturbance, tension, fatigue, vigour, and general stress and recovery significantly improved after taper compared with intense training. Sport-specific stress and recovery significantly improved after taper compared with intense training and normal training. Specific improvements in emotional stress, lack of energy, injury, emotional exhaustion, physical complaints (stress subscales), success, physical recovery, general well-being and social recovery (recovery subscales) after taper compared with intense training. Recovery subscale sleep quality significantly worse after taper compared with intense training. Across the whole study duration, general soreness, readiness to train and overall wellness were positively correlated with CMJ height. Perceptions of motivation and readiness to train were negatively correlated with distance per minute during taper. Readiness to train was positively correlated with CMJ height and negatively correlated with sprint time. Vigour was negatively correlated with sprint time. Sport-specific stress was positively correlated with player load and negatively correlated with high-speed running distance. General soreness was negatively correlated with high-speed running distance. Perceptions of tension, general soreness, anger, sRPE and total mood disturbance were negatively correlated with Wattbike PP. Anger (POMS) was positively correlated with sRPE. Tension (POMS) was negatively correlated with CMJ height. Readiness to train and general stress were negatively correlated with sprint time. General recovery and sport-specific recovery were negatively correlated with distance, distance per minute, player load and player load per minute
Dobson et al. [40]	Quantitative, longitudinal experiment without a control group with 13 collegiate swimmers	ABQ, RESTQ-Sport-52	Sport devaluation, reduced accomplishment, general stress and emotional exhaustion were significantly worse after taper compared with baseline. Self-efficacy significantly worse after taper compared with baseline and overload training
Pritchard et al. [68]	Mixed-method, open and closed survey questions with 72 elite CrossFit athletes	Tapering practices of CrossFit athletes	When asked why they taper, CrossFit athletes highlighted psychological readiness and psychological recovery as key factors. For example, one CrossFit athlete said “To mentally prepare for competition. I want to feel fresh during competition”. Another athlete said, “To ensure my body and mind is fully recovered”

*ABQ* Athlete Burnout Questionnaire, *ACRISS* Action Crisis Scale, *BRUMS* Brunel Mood Scale, *DALDA* Daily Analysis of Life Demands-Athletes, *FAS* Felt Activation Scale, *FS* Feeling Scale, *FSS* Short Flow State Scale, *LF/HF* low-frequency/high-frequency,* POMS* Profile of Mood State, *RESTQ-Sport-52* Recovery-Stress Questionnaire-Sport-52 item, *RESTQ-Sport-76* Recovery Stress Questionnaire- Sport-76 item, *RPE* Rating of Perceived Exertion, *SRSS* Short Recovery-Stress Scale, *STAI* State-Trait Anxiety Inventory

#### Mood

Mood was the most studied psychological construct in the identified articles (*n* = 20). Most articles (*n* = 18) measured mood via the Profile of Mood States (POMS) [69], a 65-item questionnaire measuring anger, confusion, depression, fatigue, tension and vigour. Alongside the individual subscales, a total mood disturbance score is also typically calculated. In addition to the Profile of Mood States, two studies used the Brunel Mood Scale [70], a shortened (24-item) version of the Profile of Mood States utilising identical mood dimensions.

Most (85%) research shows mood improves or returns to baseline levels following taper. This effect is consistent across research designs (e.g. experimental or field based), sports (e.g. swimming, triathlon, canoeing, Australian rules, rowing and cycling), and competitive levels (e.g. regional, trained, university, professional, international and elite) [43, 44]. Improvements in total mood disturbance following taper is mostly due to increases in vigour and decreases in fatigue [55], likely mirroring improvements in physiological fatigue and recovery (or cognitive appraisals of these).

A minority of research reported unchanged (5%) or deteriorated (10%) mood following taper [20, 24]. Unchanged mood could be related to training load being reduced too much during taper leading to a reduction in fitness [20], overtraining prior to taper leading to significant psychophysiological disruption, which is not reduced during taper [47], or a lack of aerobic exercise leading to increased depression or exercise addiction causing withdrawal [24]. However, an alternative psychological explanation could be that athletes are negatively appraising their performance capabilities during taper, therefore leading to disrupted mood [71].

Mood scales other than vigour and fatigue (i.e. Anger, Confusion and Depression) remain relatively stable throughout taper [25, 45]. Exceptions to this is the subscale tension, which can remain elevated following taper [24, 45]. For instance, despite total mood disturbance significantly improving following a 28-day taper, tension was found to be significantly elevated above baseline in 22 female collegiate and university swimmers [45]. Elevated tension could be due to anxiety related to the upcoming competition [31]. Another possible explanation is differences in levels of athletic experience influencing symptom appraisal. For example, less experienced athletes may lack the emotional regulation skills needed to positively appraise physiological symptoms [72]. Consequently, less experienced athletes may be more likely to appraise physiological symptoms as tension, rather than a potentially similar psychophysiological symptom such as excitement.

Some research suggests individual differences influence mood responses during taper [31, 54]. For example, a cross-sectional study found trait anxiety to influence the types and intensity of certain mood responses during taper. Specifically, significant group differences (i.e. high vs low trait anxiety) or group × time interactions were found for anger, depression, tension, confusion and vigour [54]. However, as this study used the State Trait Anxiety Inventory [73], which is a unidimensional measure, it is unknown whether cognitive or somatic anxiety exerts a greater influence on mood responses during taper. Another potentially important individual difference is gender. For example, another cross-sectional study found gender to consistently influence tension scores, with female individuals scoring higher than male individuals during taper [31]. Additionally, gender also influenced vigour and confusion scores across certain swim seasons during taper. Overall, these findings suggest trait anxiety and gender may influence the taper-mood relationship. However, further experimental or longitudinal research is required to confirm the nature and robustness of these findings.

#### Perception of Effort

Eleven articles measured perception of effort-related constructs during taper. The most used scale was the Rating of Perceived Exertion (RPE; *n* = 10), or a derivative. The RPE [74] is a subjective evaluation of physical task difficulty [75], usually measured on a 6–20 scale before and after taper. Other scales used to measure perception of effort-related constructs include the Form Scale [76], Feeling Scale [77], Felt Arousal Scale [78], Action Crisis Scale [79] and short Flow State Scale [80], each of which was used once.

Research suggests perception of effort, as measured via RPE, remains unchanged (62%) or improves (38%) following taper [17, 46]. Improvements in RPE following taper have been attributed to physiological recovery and an increased tolerance of higher intensity training [10]. For instance, research has found the RPE-power relationship to significantly improve following taper, suggesting a given power output post-taper resulted in a lower RPE compared with pre-taper [10]. Comparatively, unchanged RPE following taper could be due to low levels of pre-taper fatigue, persistent fatigue during taper or increased motivation due to positive appraisals of recovery, consequently leading to increased effort (and subsequent RPE) during training [10, 17, 19].

In addition to changes in RPE, other perception of effort-related constructs can improve during taper [39, 41]. For example, significant improvements in perceived form (i.e. the perceived current performance level of the individual), energy and feeling (i.e. how “heavy” or “light” limbs felt) were found with 25 collegiate swimmers after taper [41]. In another study, local runners doing a short-term 2-day taper had significantly less perceived physical strain, negative valence (i.e. negative arousal) and perceived action crises (i.e. conflict between continuation in achieving ones goals and task disengagement) and significantly more flow states compared with participants taking part in a lactate accumulation test [39]. Overall, these findings suggest taper enhances proprioception and facilitates the development of more positive arousal, task engagement and flow states. However, given the limited research in this area, future research needs to confirm the generalisability of these findings. Equally, the study by Venhorst et al. [39] used a relatively untrained sample and an unsupervised short-term (2-day) taper. Consequently, the ecological validity of these findings in relation to trained athletes undergoing a competition taper is questionable.

#### Perceived Fatigue and Wellness

Nine articles measured perceived fatigue and wellness, using a variety of scales. One commonly used and/or adapted scale is an eight-item questionnaire developed from recommendations by Hooper and Mackinnon [81] measuring perceptions of training, sleep, leg pain, infection, concentration, efficacy, anxiety, irritability and general stress [48, 60, 61]. With this measure, a cumulative total score of fatigue is also calculated. Other researchers have used three- [57], five- [33] or seven-item questionnaires [16] measuring similar constructs, or measured perceived well-being of the legs [42, 63]. To the authors’ knowledge, the psychometric properties of perceived fatigue and wellness questionnaires have not been formally examined (e.g. exploratory and confirmatory factor analyses). Consequently, the validity and reliability of these measures is unclear.

Most (78%) articles show perceived fatigue and wellness improve after taper [42, 61]. However, two articles (22%) reported unchanged perceived fatigue and wellness scores following taper. Low participant sizes and use of overload training phases may have underpowered the statistical test or overtrained participants, respectively, therefore explaining the unchanged perceived fatigue and wellness scores [16, 57].

Research has also found fatigue and wellness to be correlated with physiological variables [48, 60]. For instance, changes in the total score of fatigue from intense training to taper were moderately negatively correlated (*r* = −0.58) [82] with high-frequency heart-rate variability and moderately positively correlated (*r* = 0.64) with the low-frequency: high-frequency ratio in 13 (male = 9, female = 4) national-international swimmers [48]. In another study, the total score of fatigue was moderately positively correlated (*r* = 0.61) with the cortisol:cortisone ratio in 16 male elite Rugby Sevens players [60]. These findings suggest improvements in perceived fatigue and wellness during taper are associated with increased parasympathetic influence of the autonomic nervous system and reduced physiological stress.

#### Recovery-Stress Balance

Eight articles measured recovery-stress using the Recovery-Stress Questionnaire-Sport [83], or its derivatives [84]. Recovery-stress balance is theoretically grounded in a biopsychosocial conceptualisation of athlete fatigue and recovery, therefore distinguishing it from other themes in this review that have measured subjective fatigue (e.g. Perceived Fatigue and Wellness) but are not theoretically based. Most (63%) articles used the original RESTQ-Sport, which is a 76-item, 19-factor questionnaire measuring perceived stress and recovery. The 19 subscales are hierarchically organised into seven general stress subscales (general stress, emotional stress, social stress, conflicts/pressure, fatigue, lack of energy, physical complaints), five general recovery subscales (success, social recovery, physical recovery, general well-being, sleep quality), three sport-specific stress subscales (disturbed breaks, emotional exhaustion, injury) and four sport-specific recovery subscales (being in shape, personal accomplishment, self-efficacy, self-regulation). Researchers have also used shorter 52-item (25%) and eight-item (12%) versions [40, 66].

Most (88%) research shows that stress decreases and recovery increases after taper [9, 62]. Of this research, 71% used an experimental design, but often without a control group (66%). Consequently, the research lacks ecological validity and the influence of confounding and extraneous variables cannot be excluded. Only one article reported unchanged or increased stress/reduced recovery after taper [40]. This article also found that burnout dimensions of sport devaluation and reduced accomplishment increased after taper, compared with baseline. No explanation for the contradictory nature of these findings compared with other taper studies was offered; however, they could be due to the length of taper reported in the study (i.e. a 10-week period, which is substantially longer than most articles in this review). Consequently, this extensive taper period could have led to detraining [85], which may have negatively impacted perceived stress, recovery and burnout.

#### Taper as a Stressor

Six qualitative studies have findings suggesting taper may be a stressor for athletes and coaches [18, 22, 35, 51, 58, 67]. For example, transitioning from taper to fitness training was highlighted as a source of burnout in 15 professional Rugby Union players [35]. Additionally, when interviewing international athletes about competitive and organisational stressors, Hanton et al. [22] found that athletes wanted to have a good start at competition as this told them their taper was “right”. These findings suggest athletes consider taper an important performance indicator for their upcoming competition, and that when done poorly, it may be perceived to be a stressor.

Similar findings have been reported by coaches [18, 51]. In one study, 12 world class coaches (male = 6, female = 6) reported the preparation phase for major events as a challenge [51]. Specifically, one coach noted that “making sure the taper is right” (p. 453) was a stressor when preparing athletes for competition. Additionally, coaches also noted that managing athletes psychologically becomes more challenging closer to competition, as they do not behave or think in the same way they normally do [51]. Extending this, another study identified specific challenges coaches face during taper, including being negatively affected by the environment and having to maintain their composure and emotions around athletes [18]. Taken together, these findings suggest coaches face personal and interpersonal challenges specific to taper.

There is some evidence that coach and athlete behaviours change during taper, potentially owing to the stressors they experience [58, 67]. For example, seven elite athletes reported using self-compassion during taper as a coping mechanism to manage self-criticisms, prevent rumination and enhance self-belief [67]. These findings suggest athletes can be susceptible to having negative performance-related thoughts and that coping mechanisms, such as self-compassion, may be beneficial in managing these. In another study looking at the dark side of leadership behaviours in sport (i.e., Machiavellianism, psychopathy and narcissism), 15 elite male managers, head coaches and/or performance directors reported stopping using dark side behaviours during taper [58]. Although no explanation for this was provided, it could be to not “rock the boat” close to competition, possibly to reduce uncertainty and stress experienced by athletes and coaches. Like the previous theme (Psychological Preparation), further taper-specific research is required to understand the sources and impact of stress during taper.

#### Stress Tolerance

Four articles were identified measuring stress tolerance during taper. Stress tolerance was measured using The Daily Analysis of Life Demands for Athletes (DALDA). The DALDA is a 34-item questionnaire consisting of two parts: sources of stress (e.g. training and exercise) and symptoms of stress (e.g. unexplained aches) [86] in relation to training. Like perceived fatigue and wellness questionnaires, the psychometric properties of the DALDA have never been examined and therefore its validity and reliability are unclear.

Most (75%) research shows that sources of stress remain unchanged while symptoms of stress decrease [36, 49]. For example, symptoms of stress were significantly reduced in 16 male national-international triathletes following a 4-week progressive overload and a 2-week taper. Furthermore, performance on a 3-km running time trial had a weak positive correlation (*r* = 0.30) [82] with “worse than normal” responses to symptoms of stress [49]. Overall, these findings suggest that during taper, athletes experience the same stressors but at a weaker intensity, or that they are more effectively coping with the symptoms. However, most (75%) of this research used an experimental design, thus further research needs to be conducted in applied settings to confirm these findings.

Researchers have also examined the relationship between stress tolerance and physiological variables during taper, finding mixed results [36, 65]. For example, a weak negative correlation (*r* = −0.20) [82] was found between parasympathetic (lnRMSSD) heart-rate variability and stress tolerance during taper with 16 soccer players [65]. However, a moderate positive correlation (*r* = 0.41) was found between day-to-day fluctuations in parasympathetic heart-rate variability activity (lnRMSSD_cv_). Additionally, a different study found a strong positive correlation (*r* = 0.71) between symptoms of stress and cortisol and a moderate negative correlation (*r* = −0.68) between symptoms of stress and the testosterone-cortisol ratio in ten nationally ranked tennis players [36]. However, this relationship was found for the whole study duration (6 weeks of pre-season that included 1 week of taper), rather than for taper specifically. Mixed findings could be due to poor reliability or validity of the DALDA, certain physiological variables being more closely related to changes in DALDA scores than others (e.g. hypothalamic pituitary axis activation vs sympathetic-parasympathetic nervous system) or inter-sport differences in training load.

#### Psychological Preparation

Two qualitative [18, 37] and mixed-method studies [64, 68] have findings suggesting taper helps prepare athletes psychologically for competition. For example, one qualitative study reported that an elite athlete recognised they were in the optimal psychological state (defined as automatic and successful skill execution and performance feeling effortless) during taper [37]. However, what this optimal psychological state looks like during taper for different individuals and across contexts (e.g. different sports) is unknown and likely to be key to ensuring performance is maximised. Similarly, in a different qualitative study with seven Olympic and Paralympic track and field coaches, instilling confidence, coach honesty, appropriate praise, being there for the athlete and mutual confidence were identified as important factors in ensuring athletes were psychologically prepared during taper [18]. Overall, these findings suggest taper is a period in which athletes become psychologically ready to compete. However, as this research used retrospective interviews that may be susceptible to recall bias, further research is required to confirm their importance and performance impact.

Two mixed-method studies support the argument that taper is important for psychological preparation. For example, using a survey design, 72 (male = 33, female = 39) CrossFit athletes were asked (among other questions) why they taper, with athletes highlighting psychological readiness and recovery as key factors [68]. Meanwhile, a survey of 454 regional-professional strongman athletes [64] again identified psychological readiness as an important reason for engaging in taper. Taken together, these findings suggest athletes taper to be psychologically ready for competition. However, given the sport specificity and retrospective self-report nature of both surveys, whether the findings are valid across sport contexts is unknown.

#### Cognitive Functioning

One article [53] measured cognitive functioning via a computerised Stroop word colour test [87]. During a Stroop word colour test, participants must quickly and correctly identify the colour of unrelated words (i.e. the written words were not related to the colour of the ink the words were written in). Cognitive functioning was found to improve in 11 male provincial endurance athletes following a 2-week training overload and a 1-week taper [53]. Although these findings suggest taper improves cognitive functioning, the experimental nature of the study combined with the small sample size and relatively low competitive level of participants means the research has poor ecological validity.

## Discussion

This scoping review had three aims. First, to determine the characteristics of psychological research examining taper. Second, to summarise psychological research examining taper conducted with athletes and coaches. Third, to identify gaps in psychological research examining taper and suggest areas of future research. The following sections summarise the characteristics and findings of psychological research examining taper and identify gaps and future areas of research.

Most identified articles were published in the last 15 years across sports medicine, sports science, strength and conditioning, and applied sport psychology journals. Most articles identified were quantitative, using a longitudinal experimental or field study design and male athletes competing at a university, national or regional level. Of the articles identified, the most studied sport was swimming and the most common taper durations were 7 (*n* = 10), 14 (*n* = 8) or 21 days (*n* = 6). Eight themes were developed to summarise psychological research examining taper: Mood, Perception of Effort, Perceived Fatigue and Wellness, Recovery-Stress Balance, Taper as a Stressor, Stress Tolerance, Psychological Preparation and Cognitive Functioning. Research across these themes suggests taper is associated with improvements in mood, perception of effort, perceived fatigue and wellness, recovery-stress, symptoms of stress and cognitive functioning [9, 46, 49, 53, 61]. However, findings also suggest contextual and individual differences are important in influencing psychological outcomes during taper. For example, gender and trait anxiety may influence mood responses, tension can remain elevated during taper (possibly indicative of performance anxiety) and changes in the perception of effort may vary across sports [10, 45, 54]. Furthermore, qualitative and survey-based research offers novels insight into the psychology of taper, by suggesting it may be a unique stressor for athletes and coaches and that it is used as a specific psychological preparation tool [18, 22, 51, 68]. Cumulatively, this review suggests taper has multi-faceted psychological effects on athletes and coaches that are influenced by contextual and individual variables. However, despite this review highlighting the breadth and depth of psychological research examining taper, there are several limitations associated with the current evidence base and, as a consequence, many questions remain unexamined.

Results from the current study suggest, despite being studied for 30 years, the psychology of taper is under-researched. Indeed, few psychological constructs have been examined and most psychological research has been published in either sports medicine or strength and conditioning journals. This suggests that developing psychological knowledge has often not been the main aim of researchers. This is supported by researchers choice of measures, which are generally consistent with that of training monitoring rather than psychology [88]. The overall consequence of this is a superficial understanding of athletes and coaches psychological functioning during taper that prevents coaches and practitioners from integrating psychological knowledge into the planning and implementation of taper. Because of this, exploratory research is needed that explicitly examines the psychology of taper and critically discusses these findings in relation to existing psychological theory and empirical research.

There are two key areas where researchers could conduct exploratory research examining the psychology of taper. First, they could develop an inductive understanding of the psychology of taper by using semi-structured interviews to explore athletes’ and coaches’ psychological experience. Such research could be key in identifying salient psychological variables associated with taper, which could then act as a catalyst for further research. Second, they could build upon existing novel findings synthesised by this review. For example, researchers could examine confidence, the coach-athlete relationship, stress, and the multi-dimensional nature of state and trait anxiety, as these are novel constructs in taper research, but have been studied extensively in sport psychology [89]. For example, researchers could identify sources of confidence or stress during taper in athletes and coaches using semi-structured interviews or focus groups. Additionally, researchers could longitudinally examine how confidence, anxiety and performance fluctuate before and during taper leading into a real competition.

Most psychological research identified by this scoping review examines basic questions, such as whether variables change from pre- to post-taper. Although this research is useful in showing how taper influences these variables, this type of research is reductionistic in reducing the potentially complex psychological experience of taper. Indeed, many psychological questions remain unanswered, such as how psychological variables fluctuate during taper, how psychological variables interact and influence one another during taper, and how pre-taper psychological states influence during taper psychological states. Because of these limitations, researchers could build upon existing research by asking more advanced research questions. For example, researchers could study what mediates or moderates the relationship between psychological variables, such as mood or recovery-fatigue balance, and post-taper performance. Specifically, researchers could examine whether social (e.g. coach-athlete relationship, group cohesion, teamwork) or individual differences (e.g. personality) interact with the relationship between taper and mood (or other already-measured variables) or post-taper performance. Examining the interaction or association between physiological and psychological variables during taper (i.e. conducting multidisciplinary research) may also be fruitful avenues for research. For example, interpretations of physiological states are an antecedent of self-efficacy [71], which directly influences athletic performance [90]. In turn, successful athletic performance further enhances self-efficacy, therefore causing a positive feedback loop [71]. Consequently, it is likely interpretations of physiological changes during taper have psychological and performance implications. To examine this, future researchers could seek to develop a theoretically informed model of the relationship between fatigue/recovery, strength, power, self-efficacy, and performance and then examine this with athletes during taper. Similarly, little is known about how perceived fatigue monitoring can be used to make real-time refinements to taper, and what impact this has on performance and athletes’ optimal psychological states. Research could therefore develop and test a protocol that uses perceived fatigue to alter training load and examine the impact this has on psychological variables (e.g. confidence) and performance.

Psychological research identified by this scoping review has focused predominantly on male athletes, with one study undertaken with coaches [18] and none with disabled athletes. This represents a significant gap in knowledge, as gender has been shown to influence psychological variables during taper [54], coaches may be susceptible to stress during taper [51], and tapering effectively is important in disability sports as athletes may be susceptible to overtraining or training inconsistently [91]. Consequently, there is a clear need for the inclusion of more diverse populations within this research area. Researchers could therefore quantitatively examine whether gender moderates how athletes psychologically respond to taper. Equally, researchers could qualitatively explore coaches understanding of taper. Finally, researchers could conduct exploratory research examining taper in disability sports, by studying what taper means physiologically and psychologically to disabled athletes and how it is implemented by coaches.

No intervention research was identified by this review, and therefore the effectiveness and efficacy of strategies for enhancing performance and psychological outcomes with adult athletes during taper remain unknown. The lack of intervention research is likely owing to a lack of understanding around what psychological variables are important during taper, and therefore psychological research needs to work towards building an evidence base that can be used to implement interventions to improve psychological states and/or sport performance during taper. Consequently, future research should begin examining the impact of psychological interventions during taper, potentially using findings from the aforementioned future research directions as a  theoretetical foundation. Specifically, using a single case design, researchers could explore the impact of self-efficacy or biofeedback-based interventions on athletes’ performance and psychological state prior to, or during, taper. Equally, researchers could implement interventions targeting psychological variables moderating or mediating the relationship between taper and performance at the team or organisational level (e.g. coach-athlete communication, motivational climate).

### Limitations

This scoping review has several limitations. For example, we only included articles that used an adult sample, meaning we may have excluded insightful research using an under 18 years of age sample. However, we only wanted to review adult research to provide a specific understanding of the current research base given the developmental differences between adults and adolescents [27]. Another limitation was the elements of our search protocol. For example, we only searched at the title, abstract, keyword and subject heading (where allowed) level. This was done to ensure searching was consistent across the different databases; however, using a different protocol (e.g. searching using all database tags) may have returned different results. Despite this, our use of subject heading-based searches, alongside alternative searching strategies (i.e. reference lists, “cited by” searches and manual journal searches), would have likely mitigated against this. A final limitation of this review is its lack of systematic quality appraisal, therefore meaning the quality of the reviewed research is unknown. The lack of a systematic quality appraisal is consistent with scoping review recommendations and aims, whereby the aim is not to determine the quality of evidence but rather to map the research area [13]. However, where appropriate we have evaluated evidence to provide readers with a critical understanding of research findings.

## Conclusions

This study represents the first rigorous and transparent review of psychological research examining taper and significantly contributes to the literature in three ways. First, this is the first review to highlight the key characteristics of psychological research examining taper, therefore offering novel insight into the types of research being conducted and who it is being conducted with. Second, this review builds upon and extends existing understanding of psychological research examining taper [4] by providing an up-to-date and critical summary of current and emerging psychological research examining taper. Third, this is the first review to consider the literature as a whole and offer future research directions to advance the field. Overall, this scoping review has highlighted the lack of research examining the psychology of taper and the need for focused research that asks more complex questions across diverse populations.

## Supplementary Information

Below is the link to the electronic supplementary material.

Supplementary file1 (DOCX 43 kb)

Supplementary file2 (PDF 158 kb)

Supplementary file3 (XLSX 61 kb)

## Data Availability

Database search results are available upon request from the lead author.
