# Correction to: The Psychology of Athletic Tapering in Sport: A Scoping Review

**DOI:** 10.1007/s40279-023-01824-1

**Published:** 2023-02-18

**Authors:** Maxwell J. Stone, Camilla J. Knight, Ross Hall, Catherine Shearer, Ross Nicholas, David A. Shearer

**Affiliations:** 1 School of Psychology, Newcastle University, Newcastle upon Tyne, UK; 2 Department of Sport and Exercise Sciences, Swansea University, Swansea, UK; 3 Welsh Institute of Performance Science, Sport Wales Institute, Cardiff, UK; 4 Faculty of Life Sciences and Education, University of South Wales, Pontypridd, UK; 5 Sport Wales Institute, Cardiff, UK; 6 Swim Wales, Swansea, UK; 7 Department of Sport Science and Physical Education, University of Agder, Kristiansand, Norway

**Correction to: Sports Medicine** 10.1007/s40279-022-01798-6

Following online publication, some numerical errors were identified in paragraph 3 of the section entitled, “3.2 Characteristics of Psychological Research Examining Taper”.

This paragraph, which previously read:

Most (92%) articles used an athlete sample (*n* = 44), with only three articles examining coaches and one article examining both athletes and coaches. In total, the articles included 1531 athletes, of which 1062 (69%) were male and 496 were female (31%). Three studies [17, 35, 36], equating to 30 participants, did not report or make explicit the gender of the participants. A total of 34 coaches were included in studies (28 were male and six were female). In one article using both an athlete and coach sample, 15 were female and eight were male [37]. The gender of the individual athlete and coach groups was not reported.

Has now been updated to read:

Most (92%) articles used an athlete sample (n = 44), with only three articles examining coaches and one article examining both athletes and coaches. In total, the articles included 1588 participants. Of these participants, 1548 were athletes (97%) and 40 were coaches (3%). Of the athletes, 1026 were male (66%) and 475 were female (31%). Of the coaches, 28 were male (70%) and 6 were female (15%). Across four studies [17, 35, 36, 37], the gender of 47 athletes and six coaches was not reported or made explicit (representing 3% and 15% of the respective total percentages).

These errors also affected the numbers reported in Table 2. Table 2 has therefore also been updated.

**Table 2:** Study characteristics of psychologically related taper research

Study characteristic	*n*	%
**Publication year**		
1989–1994	6	13
1995–2000	4	8
2001–2006	4	8
2007–2012	9	19
2013–2018	17	35
2019–present	8	17
**Journal**		
Journal of Strength and Conditioning Research	6	13
Medicine and Science in Sports and Exercise	6	13
International Journal of Sports Medicine	5	10
Journal of Applied Sport Psychology	4	8
Journal of Sports Sciences	4	8
Scandinavian Journal of Medicine and Science in Sports	3	6
Biology of Sport	2	4
International Journal of Sports Science and Coaching	2	4
Journal of Science and Medicine in Sport	2	4
Applied Physiology, Nutrition, and Metabolism	1	2
Canadian Journal of Applied Physiology	1	2
European Journal of Sport Science	1	2
International Journal of Sport and Exercise Psychology	1	2
International Journal of Sports Physiology and Performance	1	2
International Journal of Wrestling Science	1	2
Journal of Human Kinetics	1	2
Journal of Sports Medicine and Physical Fitness	1	2
Journal of the American College of Nutrition	1	2
Perceptual and Motor Skills	1	2
Psychology of Sport and Exercise	1	2
Psychoneuroendocrinology	1	2
Sports Medicine—Open	1	2
The Sport Psychologist	1	2
**Design**		
Quantitative	38	79
Qualitative	7	15
Mixed method	3	6
**Methodology**		
Longitudinal experiment with control group	11	23
Longitudinal field study	11	23
Longitudinal experiment without control group	9	19
Not reported/explicit	5	10
Case study	2	4
Longitudinal quasi-experiment	2	4
Survey	2	4
Cross-sectional field study	1	2
Cross-sectional quasi-experiment	1	2
Interpretive constructionist	1	2
Longitudinal field study with experimental (no control group) follow up	1	2
Longitudinal field study with follow up focus groups	1	2
Pragmatism	1	2
**Participant**		
Athlete	44	92
Coach	3	6
Athlete and coach	1	2
**Participant gender**		
Male	1062	67
Female	496	31
Not reported/explicit	30	2
**Participant descriptor**		
Elite	13	27
University	12	25
Regional	7	15
National	6	13
Professional/competitive	3	6
Trained	3	6
High-level	2	4
International	2	4
**Participant sport**		
Swimming	11	23
Multisport	7	15
Triathlon	6	13
Cycling	5	10
Rugby	3	6
Running	2	4
Australian Rules	1	2
Canoeing	1	2
CrossFit	1	2
Endurance sport	1	2
Mountain biking	1	2
Not reported/explicit	1	2
Rowing	1	2
Soccer	1	2
Strongmen	1	2
Tennis	1	2
Track and field	1	2
Water-polo	1	2
Weightlifting	1	2
Wrestling	1	2
**Taper duration (days)**		
2	1	2
7	10	21
10	2	4
14	8	17
21	6	13
28	4	8
70	1	2
n/a	9	19
Not reported/explicit	5	10
Multiple	2	4
**Psychological measure/data collection method**		
***Mood***		
Profile of Mood State	18	26
Brunel Mood Scale	2	3
***Perceived exertion***		
Rating of Perceived Exertion	10	14
Action Crisis Scale	1	1
Feeling Scale	1	1
Felt Arousal Scale	1	1
Form Scale	1	1
Short Flow State Scale	1	1
***Perceived wellness and fatigue***		
Fatigue, sleep quality, muscle soreness, stress levels, and mood Perceived wellbeing in the legs	2	3
Training, sleep, leg pain, infection, concentration, efficacy, anxiety, irritability, and general stress	2	3
Mood state, energy levels, stress, fatigue, and muscle soreness	2	3
Pain, recovery, and fatigue	1	1
Sleep quality, readiness to train, general muscular soreness, fatigue, stress, mood, and motivation	1	1
***Recovery-stress***		
Recovery-Stress Questionnaire-Sport (72 item)	1	1
Recovery-Stress Questionnaire-Sport (52 item)	6	9
Short Recovery Stress Questionnaire	1	1
***Stress tolerance***		
Daily Analysis of Life Demands	4	6
***Cognitive functioning***		
STROOP task	1	1
***Qualitative***		
Semi-structured interviews	7	10
Focus groups	1	1
***Survey***		
Open and closed questions asking about tapering practices	2	3
***Other***		
Athlete Burnout Questionnaire	1	1
State-Trait Anxiety Inventory	1	1

Due to rounding some total percentages do not equate to 100%

The original article has been corrected.

