# Supplementary material for: The Psychology of Athletic Tapering in Sport: A Scoping Review
Source: Sports Med. 2023 Jan 25;53(4):777–801. doi: 10.1007/s40279-022-01798-6 (PMC10036416; doi:10.1007/s40279-022-01798-6)
Supplement: Supplementary file 1 — Supplementary file1 (DOCX 43 kb) [file 40279_2022_1798_MOESM1_ESM.docx]

**The Psychology of Taper: A Scoping Review**

Sports Medicine

Maxwell J. Stone^1^, Camilla J. Knight^2, 3^, Ross Hall^4^, Catherine Shearer^5^, Ross Nicholas^6^, David A. Shearer^3, 4^

^1^Newcastle University, School of Psychology, Newcastle upon Tyne, United Kingdom; ^2^Swansea University, School of Engineering and Applied Sciences, Swansea, United Kingdom; ^3^Welsh Institute of Performance Science, Sport Wales Institute, Cardiff, United Kingdom; ^4^University of South Wales, Faculty of Life Sciences and Education, Pontypridd, United Kingdom; ^5^Sport Wales Institute, Cardiff, United Kingdom, ^6^Swim Wales, Swansea, United Kingdom

Correspondence concerning this article should be addressed to: Maxwell. J. Stone, Newcastle University, Newcastle upon Tyne, UK, NE2 4DR, +44 190 2086176, [max.stone@newcastle.ac.uk](mailto:max.stone@newcastle.ac.uk)

**Online Resource 1** Scoping Review Search Strategy

**Research objectives**

1. Identify and summarise the key study characteristics of psychological research examining the taper
2. Summarise and critically evaluate the key areas of psychological research examining the taper
3. Identify future areas of psychological research into the taper

**Overall search strategy**

1. Searching of electronic databases
   1. Subject heading, title, abstract, and keywords
2. Use of existing knowledge/networks to identify relevant resources
3. Manual searching of journals
   1. International Review of Sport and Exercise Psychology, Psychology of Sport and Exercise, Sport Exercise and Performance Psychology, Journal of Sport and Exercise Psychology, Journal of Applied Sport Psychology, International Journal of Sport and Exercise Psychology
4. After screening:
   1. Searching of reference lists of relevant articles
   2. Cited searches of relevant articles

| **Suggested databases** | |
| --- | --- |
| **Database** | **Coverage** |
| Scopus | Multidisciplinary; science, technology, medicine, social sciences, and arts and humanities |
| Web of Science | Multidisciplinary |
| PsycARTICLES & PsycINFO | Psychology; multidisciplinary research in psychological, social, behavioural, and health sciences |
| SportDiscus | Sport science; nutrition, physical therapy, occupation health and therapy, exercise physiology, and kinesiology |
| PubMed | Biomedicine and health fields: life sciences, behavioural sciences, chemical sciences, and bioengineering (including Medline, PubMed Central, and Bookshelf) |

**Key search terms and related terms**

- Taper* - tapering, tapered, taper
- Psych* – psychology, psychological, psychologically
  - Mental* - mental, mentality
  - Mental skills
  - Cogniti* - cognitions, cognition, cognitively
  - Emoti* - emotions, emotion, emotional, emotive
  - Behav* - behaviour, behaviours, behaviourally
- Sport* - sport, sporting
  - Recover* - recovery, recover
  - Fatigu* - fatigue, fatigued, fatiguing
  - Prepar* - preparation, preparing, prepared
  - Peak* - peaking, peak, peaked
  - Optim* - optimal, optimisation
  - Pre-perform* - pre-performance, pre-performed, pre-performing
  - Perform* - performance, performed
  - Compet* - compete, competition, competitive
  - Train* - training, trained
  - Athlet* - athletes, athletic, athlete
  - Coach* - coach, coaches, coached

|  | **Pilot searches** | |
| --- | --- | --- |
| **Search number** | **Search term** | **Hits and notes** |
| 1 | taper* AND (psych* OR mental* OR “mental skills” OR cogniti OR emoti* OR behave*) AND (sport* OR recover* OR fatigue* OR prepar* OR peak* OR optim* OR pre-perform* OR perform* OR compet* OR train* OR athlet* OR coach*) | Scopus (title, abstract, keywords) – 2519 hits.  Perform* and optim* appears to be returning hits relating to drug therapy. Consequently, these terms will be removed. |
| 2 | taper* AND (psych* OR mental* OR “mental skills” OR cogniti OR emoti* OR behave*) AND (sport* OR recover* OR fatigue* OR prepar* OR peak* OR pre-perform* OR compet* OR train* OR athlet* OR coach*) | Scopus (title, abstract, keywords) – 1126 hits.  Prepar* returning engineering and drug related hits. Consequently, this term will be removed. |
| 3 | taper* AND (psych* OR mental* OR “mental skills” OR cogniti OR emoti* OR behave*) AND (sport* OR recover* OR fatigu* OR peak* OR pre-perform* OR compet* OR train* OR athlet* OR coach*) | Scopus (title, abstract, keywords) – 935 hits.  Peak* returning engineering hits. This term will be removed. |
| 4 | taper* AND (psych* OR mental* OR “mental skills” OR cogniti OR emoti* OR behave*) AND (sport* OR recover* OR fatigue* OR pre-perform* OR compet* OR train* OR athlet* OR coach*) | Scopus (title, abstract, keywords) – 742 hits.  Looks much more concentrated. Recover* fatigue*, and train* returning some irrelevant hits but will be retained given their relevance to the research question and the fact they are returning positive hits. |

| **Inclusion/exclusion criteria** | | |
| --- | --- | --- |
| **Criteria no.** | **Criteria** | **Rationale** |
| 1 | English only articles | Time/cost associated with translation |
| 2 | Peer reviewed articles (including in press articles) | Only interested in articles which have passed scientific scrutiny |
| 3 | Full texts must be available | Full texts are required to ensure information from them can be accurately and fairly presented |
| 4 | Must measure (i.e., quantitative) or discuss (i.e., qualitative) cognitive, emotional, or behavioural factors (i.e., psychological factors). | Only interested in psychological research |
| 5 | Research must be conducted in the context of tapering or have findings which reference the taper. We define tapering in line with Mujika and Padilla (2003) as a period of progressively reduced training aiming to reduce physiological and psychological fatigue and increase performance | Only interested in research which has been conducted during the taper/provides insights into tapering |
| 6 | In primary research studies, participants must be over 18 years of age | Over 18’s only as there are developmental, psychological, and physiological differences between adults and adolescents/children |
| 7 | In primary research studies, participants must be athletes (i.e., individuals competing in sport) or sports coaches (i.e., individuals involved in the training of athletes in sport) involved in sport (competitive physical activity requiring skill and/or physical prowess) | Only interested in sport-based research |
| **No restrictions on:** (a) sport; (b) study design (i.e., quantitative and qualitative); (c) time frame (i.e., cross sectional or longitudinal), or; (d) publication dates. | | |

**Online Resource 3** Complete search term and number of hits for each database

**Electronic Databases (N = 6) Search (27.03.21)**

| **Database/platform** | Scopus (Elsevier) |
| --- | --- |
| **Date coverage** | 1788 to present |
| **Library** | Newcastle University |
| **Date of search** | 27/03/21 |
| **Limits** | In: Article Title, Abstract, Keywords  Document type: Article, Review  Language: English |
| **Search query** | Search ID#:   1. TITLE-ABS-KEY ( taper* ) 2. TITLE-ABS-KEY ( psych* ) 3. TITLE-ABS-KEY ( mental* ) 4. TITLE-ABS-KEY ( "mental skills" ) 5. TITLE-ABS-KEY ( cogniti* ) 6. TITLE-ABS-KEY ( emoti* ) 7. TITLE-ABS-KEY ( behav* ) 8. TITLE-ABS-KEY ( sport* ) 9. TITLE-ABS-KEY ( recover* ) 10. TITLE-ABS-KEY ( fatigu* ) 11. TITLE-ABS-KEY ( pre-perform* ) 12. TITLE-ABS-KEY ( compet* ) 13. TITLE-ABS-KEY ( train* ) 14. TITLE-ABS-KEY ( athlet* ) 15. TITLE-ABS-KEY ( coach* ) 16. #2 OR #3 OR #4 OR #5 OR #6 OR #7 17. #8 OR #9 OR #10 OR #11 OR #12 OR #13 OR #14 OR 15# 18. #1 AND #16 AND #17 |
| **Number of hits** | 569 |

| **Database/platform** | Web of Science (Clarivate Analytics) |
| --- | --- |
| **Date coverage** | 1900 to present |
| **Library** | Newcastle University |
| **Date of search** | 27/03/21 |
| **Limits** | In: Topic (Title, Abstract, Author Keywords, and Author Keywords Plus)  Document type: Article, Review  Language: English |
| **Search query** | Search ID#   1. ts=(taper*) 2. ts=(psych* OR mental* OR “mental skills” OR cogniti OR emoti* OR behave*) 3. ts=(sport* OR recover* OR fatigue* OR pre-perform* OR compet* OR train* OR athlet* OR coach*) 4. #3 AND #2 AND #1 |
| **Number of hits** | 314 |

| **Database/platform** | APA PsycInfo (Ovid) |
| --- | --- |
| **Date coverage** | 1806 to present |
| **Library** | Newcastle University |
| **Date of search** | 27/03/21 |
| **Limits** | In: Abstract, Key Concepts, Title, Subject Headings (where relevant)  Publication type: Peer Reviewed Journal |
| **Search query** | Search ID#:   1. "taper*".ab,id,ti. 2. "psych*".ab,id,ti. 3. "mental*".ab,id,ti. 4. mental skills.ab,id,ti. 5. "cogniti*".ab,id,ti. 6. "emoti*".ab,id,ti. 7. "behav*".ab,id,ti. 8. "sport*".ab,id,ti. 9. "pre-perform*".ab,id,ti. 10. "recover*".ab,id,ti. 11. "fatigu*".ab,id,ti. 12. "compet*".ab,id,ti. 13. "train*".ab,id,ti. 14. "athlet*".ab,id,ti. 15. "coach*".ab,id,ti. 16. exp Cognition/ 17. exp Emotions/ 18. exp Behavior/ 19. exp Sports/ 20. exp Fatigue/ 21. exp Competition/ 22. exp Athletic Training/ or exp Training/ 23. exp Sport Psychology/ or exp Psychology/ 24. exp College Athletes/ or exp Athletes/ or exp Professional Athletes/ 25. exp Coaches/ 26. 2 or 3 or 4 or 5 or 6 or 7 or 16 or 17 or 18 or 23 27. 8 or 9 or 10 or 11 or 12 or 13 or 14 or 15 or 19 or 20 or 21 or 22 or 24 or 25 28. 1 and 26 and 27 |
| **Number of hits** | 111 |

| **Database/platform** | APA PsycArticles Full Text (Ovid) |
| --- | --- |
| **Date coverage** | 1985 |
| **Library** | Newcastle University |
| **Date of search** | 27/03/21 |
| **Limits** | In: Abstract, Title, Author Keywords  Publication type: Article |
| **Search query** | Search ID#   1. "taper*".ti,ab,kw. 2. "psych*".ti,ab,kw. 3. "mental*".ti,ab,kw. 4. mental skills.ti,ab,kw. 5. "cogniti*".ti,ab,kw. 6. "emoti*".ti,ab,kw. 7. "behav*".ti,ab,kw. 8. "sport*".ti,ab,kw. 9. "recover*".ti,ab,kw. 10. "fatigu*".ti,ab,kw. 11. "pre-perform*".ti,ab,kw. 12. "compet*".ti,ab,kw. 13. "train*".ti,ab,kw. 14. "athlet*".ti,ab,kw. 15. "coach*".ti,ab,kw. 16. 2 or 3 or 4 or 5 or 6 or 7 17. 8 or 9 or 10 or 11 or 12 or 13 or 14 or 15 18. 1 and 16 and 17 |
| **Number of hits** | 4 |

| **Database/platform** | SPORTDiscus (EBSCOhost) |
| --- | --- |
| **Date coverage** | 1892 to present |
| **Library** | Newcastle University |
| **Date of search** | 27/03/21 |
| **Limits** | In: Abstract, Title, and Author Keywords, Subjects (where appropriate)  Source Types: Academic Journals  Limit to: Peer Reviewed  Language: English |
| **Search query** | Search ID#:   1. TI taper* OR AB taper* OR KW taper* 2. TI psych* OR AB psych* OR KW psych* 3. TI mental* OR AB mental* OR KW mental* 4. TI "mental skills" OR AB "mental skills" OR KW "mental skills" 5. TI cogniti* OR AB cogniti* OR KW cogniti* 6. TI emoti* OR AB emoti* OR KW emoti* 7. TI behav* OR AB behav* OR KW behav* 8. TI sport* OR AB sport* OR KW sport* 9. TI recover* OR AB recover* OR KW recover* 10. TI fatigu* OR AB fatigu* OR KW fatigu* 11. TI pre-perform* OR AB pre-perform* OR KW pre-perform* 12. TI compete* OR AB compete* OR KW compete* 13. TI train* OR AB train* OR KW train* 14. TI athlet* OR AB athlet* OR KW athlet* 15. TI coach* OR AB coach* OR KW coach* 16. DE “PSYCHOLOGY” OR DE “PSYCHOLOGY of athletes” OR DE “MENTAL training” OR DE “COGNITION” OR DE “EMOTIONS” 17. DE “SPORTS” OR DE “RECOVERY training” OR DE “FATIGUE” OR DE “SPORTS competitions” OR DE “ATHLETE training” OR DE “ATHLETES” OR DE COACHES (athletics)” 18. S2 OR S3 OR S4 OR S5 OR S6 OR S7 OR S16 19. S8 OR S9 OR S10 OR S11 OR S12 OR S13 OR S14 OR S15 OR S17 20. S1 AND S18 AND S19 |
| **Number of hits** | 100 |

| **Database/platform** | PubMed Central (National Library of Medicine) |
| --- | --- |
| **Date coverage** | 1975 to present |
| **Library** | Newcastle University |
| **Date of search** | 27/03/21 |
| **Limits** | In: Title, Abstract, Other Term, and Text Word  Article type: Journal Article, Meta-Analysis, Systematic Review, Review  Language: English |
| **Search query** | **Search ID#**   1. **((taper*[Title/Abstract]) OR (taper*[Other Term])) OR (taper*[Text Word])** 2. **((psych*[Title/Abstract]) OR (psych*[Other Term])) OR (psych*[Text Word])** 3. **((mental*[Title/Abstract]) OR (mental*[Other Term])) OR (mental*[Text Word])** 4. **(("mental skills"[Title/Abstract]) OR ("mental skills"[Other Term])) OR ("mental skills"[Text Word])** 5. **((cogniti*[Title/Abstract]) OR (cogniti*[Other Term])) OR (cogniti*[Text Word])** 6. **((emoti*[Title/Abstract]) OR (emoti*[Other Term])) OR (emoti*[Text Word])** 7. **((behav*[Title/Abstract]) OR (behav*[Other Term])) OR (behav*[Text Word])** 8. **((sport*[Title/Abstract]) OR (sport*[Other Term])) OR (sport*[Text Word])** 9. **((recover*[Title/Abstract]) OR (recover*[Other Term])) OR (recover*[Text Word])** 10. **((fatigu*[Title/Abstract]) OR (fatigu*[Other Term])) OR (fatigu*[Text Word])** 11. **((pre-perform*[Title/Abstract]) OR (pre-perform*[Other Term])) OR (pre-perform*[Text Word])** 12. **((compet*[Title/Abstract]) OR (compet*[Other Term])) OR (compet*[Text Word])** 13. **((train*[Title/Abstract]) OR (train*[Other Term])) OR (train*[Text Word])** 14. **((athlet*[Title/Abstract]) OR (athlet*[Other Term])) OR (athlet*[Text Word])** 15. **((coach*[Title/Abstract]) OR (coach*[Other Term])) OR (coach*[Text Word])** 16. **#2 OR #3 OR #4 OR #5 OR #6 OR #7** 17. **#8 OR #9 OR #10 OR #11 OR #12 OR #13 OR #14 OR #15** 18. #1 AND #16 AND #17 |
| **Number of hits** | 526 |

| **Total** | 1624 |
| --- | --- |
| **After deduplication** | 987 |
| **Conflicts after abstract and title screening** | 52 (5.27%) |

**Manual Searches of Sport Psychology Journals (N = 7) Search (29.03.21)**

| Journal | Psychology of Sport and Exercise |
| --- | --- |
| Date | 29.03.21 |
| Search term | Taper |
| Limits | None |
| Hits | 18 |

| Journal | Journal of Sport and Exercise Psychology |
| --- | --- |
| Date | 29.03.21 |
| Search term | Taper |
| Limits | None |
| Hits | 0 |

| Journal | Journal of Applied Sport Psychology |
| --- | --- |
| Date | 29.03.21 |
| Search term | Taper |
| Limits | None |
| Hits | 17 |

| Journal | International Journal of Sport and Exercise Psychology |
| --- | --- |
| Date | 29.03.21 |
| Search term | Taper |
| Limits | None |
| Hits | 5 |

| Journal | Journal of Clinical Sport Psychology |
| --- | --- |
| Date | 29.03.21 |
| Search term | Taper |
| Limits | None |
| Hits | 1 |

| Journal | Journal of Sport Psychology in Action |
| --- | --- |
| Date | 29.03.21 |
| Search term | Taper |
| Limits | None |
| Hits | 2 |

| Journal | Sport, Exercise, and Performance Psychology |
| --- | --- |
| Date | 29.03.21 |
| Search term | Taper |
| Limits | None |
| Hits | 0 |
| **Total** | 43 |
| **After deduplication** | 38 |

**Electronic Databases (N = 6) Search (05.11.21)**

| **Database/platform** | Scopus (Elsevier) |
| --- | --- |
| **Date coverage** | 1788 to present |
| **Library** | Newcastle University |
| **Date of search** | 05/11/21 |
| **Limits** | In: Article Title, Abstract, Keywords  Document type: Article, Review  Language: English |
| **Search query** | Search ID#:   1. TITLE-ABS-KEY ( taper* ) 2. TITLE-ABS-KEY ( psych* ) 3. TITLE-ABS-KEY ( mental* ) 4. TITLE-ABS-KEY ( "mental skills" ) 5. TITLE-ABS-KEY ( cogniti* ) 6. TITLE-ABS-KEY ( emoti* ) 7. TITLE-ABS-KEY ( behav* ) 8. TITLE-ABS-KEY ( sport* ) 9. TITLE-ABS-KEY ( recover* ) 10. TITLE-ABS-KEY ( fatigu* ) 11. TITLE-ABS-KEY ( pre-perform* ) 12. TITLE-ABS-KEY ( compet* ) 13. TITLE-ABS-KEY ( train* ) 14. TITLE-ABS-KEY ( athlet* ) 15. TITLE-ABS-KEY ( coach* ) 16. #2 OR #3 OR #4 OR #5 OR #6 OR #7 17. #8 OR #9 OR #10 OR #11 OR #12 OR #13 OR #14 OR 15# 18. #1 AND #16 AND #17 |
| **Additional hits** | 23 |

| **Database/platform** | Web of Science (Clarivate Analytics) |
| --- | --- |
| **Date coverage** | 1900 to present |
| **Library** | Newcastle University |
| **Date of search** | 05/11/21 |
| **Limits** | In: Topic (Title, Abstract, Author Keywords, and Author Keywords Plus)  Document type: Article, Review  Language: English |
| **Search query** | Search ID#   1. ts=(taper*) 2. ts=(psych* OR mental* OR “mental skills” OR cogniti OR emoti* OR behave*) 3. ts=(sport* OR recover* OR fatigue* OR pre-perform* OR compet* OR train* OR athlet* OR coach*) 4. #3 AND #2 AND #1 |
| **Additional hits** | 13 |

| **Database/platform** | APA PsycInfo (Ovid) |
| --- | --- |
| **Date coverage** | 1806 to present |
| **Library** | Newcastle University |
| **Date of search** | 05/11/21 |
| **Limits** | In: Abstract, Key Concepts, Title, Subject Headings (where relevant)  Publication type: Peer Reviewed Journal |
| **Search query** | Search ID#:   1. "taper*".ab,id,ti. 2. "psych*".ab,id,ti. 3. "mental*".ab,id,ti. 4. mental skills.ab,id,ti. 5. "cogniti*".ab,id,ti. 6. "emoti*".ab,id,ti. 7. "behav*".ab,id,ti. 8. "sport*".ab,id,ti. 9. "pre-perform*".ab,id,ti. 10. "recover*".ab,id,ti. 11. "fatigu*".ab,id,ti. 12. "compet*".ab,id,ti. 13. "train*".ab,id,ti. 14. "athlet*".ab,id,ti. 15. "coach*".ab,id,ti. 16. exp Cognition/ 17. exp Emotions/ 18. exp Behavior/ 19. exp Sports/ 20. exp Fatigue/ 21. exp Competition/ 22. exp Athletic Training/ or exp Training/ 23. exp Sport Psychology/ or exp Psychology/ 24. exp College Athletes/ or exp Athletes/ or exp Professional Athletes/ 25. exp Coaches/ 26. 2 or 3 or 4 or 5 or 6 or 7 or 16 or 17 or 18 or 23 27. 8 or 9 or 10 or 11 or 12 or 13 or 14 or 15 or 19 or 20 or 21 or 22 or 24 or 25 28. 1 and 26 and 27 |
| **Additional hits** | 32 |

| **Database/platform** | APA PsycArticles Full Text (Ovid) |
| --- | --- |
| **Date coverage** | 1985 |
| **Library** | Newcastle University |
| **Date of search** | 05/11/21 |
| **Limits** | In: Abstract, Title, Author Keywords  Publication type: Article |
| **Search query** | Search ID#   1. "taper*".ti,ab,kw. 2. "psych*".ti,ab,kw. 3. "mental*".ti,ab,kw. 4. mental skills.ti,ab,kw. 5. "cogniti*".ti,ab,kw. 6. "emoti*".ti,ab,kw. 7. "behav*".ti,ab,kw. 8. "sport*".ti,ab,kw. 9. "recover*".ti,ab,kw. 10. "fatigu*".ti,ab,kw. 11. "pre-perform*".ti,ab,kw. 12. "compet*".ti,ab,kw. 13. "train*".ti,ab,kw. 14. "athlet*".ti,ab,kw. 15. "coach*".ti,ab,kw. 16. 2 or 3 or 4 or 5 or 6 or 7 17. 8 or 9 or 10 or 11 or 12 or 13 or 14 or 15 18. 1 and 16 and 17 |
| **Additional hits** | 0 |

| **Database/platform** | SPORTDiscus (EBSCOhost) |
| --- | --- |
| **Date coverage** | 1892 to present |
| **Library** | Newcastle University |
| **Date of search** | 05/11/21 |
| **Limits** | In: Abstract, Title, and Author Keywords, Subjects (where appropriate)  Source Types: Academic Journals  Limit to: Peer Reviewed  Language: English |
| **Search query** | Search ID#:   1. TI taper* OR AB taper* OR KW taper* 2. TI psych* OR AB psych* OR KW psych* 3. TI mental* OR AB mental* OR KW mental* 4. TI "mental skills" OR AB "mental skills" OR KW "mental skills" 5. TI cogniti* OR AB cogniti* OR KW cogniti* 6. TI emoti* OR AB emoti* OR KW emoti* 7. TI behav* OR AB behav* OR KW behav* 8. TI sport* OR AB sport* OR KW sport* 9. TI recover* OR AB recover* OR KW recover* 10. TI fatigu* OR AB fatigu* OR KW fatigu* 11. TI pre-perform* OR AB pre-perform* OR KW pre-perform* 12. TI compete* OR AB compete* OR KW compete* 13. TI train* OR AB train* OR KW train* 14. TI athlet* OR AB athlet* OR KW athlet* 15. TI coach* OR AB coach* OR KW coach* 16. DE “PSYCHOLOGY” OR DE “PSYCHOLOGY of athletes” OR DE “MENTAL training” OR DE “COGNITION” OR DE “EMOTIONS” 17. DE “SPORTS” OR DE “RECOVERY training” OR DE “FATIGUE” OR DE “SPORTS competitions” OR DE “ATHLETE training” OR DE “ATHLETES” OR DE COACHES (athletics)” 18. S2 OR S3 OR S4 OR S5 OR S6 OR S7 OR S16 19. S8 OR S9 OR S10 OR S11 OR S12 OR S13 OR S14 OR S15 OR S17 20. S1 AND S18 AND S19 |
| **Additional hits** | 3 |

| **Database/platform** | PubMed Central (National Library of Medicine) |
| --- | --- |
| **Date coverage** | 1975 to present |
| **Library** | Newcastle University |
| **Date of search** | 05/11/21 |
| **Limits** | In: Title, Abstract, Other Term, and Text Word  Article type: Journal Article, Meta-Analysis, Systematic Review, Review  Language: English |
| **Search query** | **Search ID#**   1. **((taper*[Title/Abstract]) OR (taper*[Other Term])) OR (taper*[Text Word])** 2. **((psych*[Title/Abstract]) OR (psych*[Other Term])) OR (psych*[Text Word])** 3. **((mental*[Title/Abstract]) OR (mental*[Other Term])) OR (mental*[Text Word])** 4. **(("mental skills"[Title/Abstract]) OR ("mental skills"[Other Term])) OR ("mental skills"[Text Word])** 5. **((cogniti*[Title/Abstract]) OR (cogniti*[Other Term])) OR (cogniti*[Text Word])** 6. **((emoti*[Title/Abstract]) OR (emoti*[Other Term])) OR (emoti*[Text Word])** 7. **((behav*[Title/Abstract]) OR (behav*[Other Term])) OR (behav*[Text Word])** 8. **((sport*[Title/Abstract]) OR (sport*[Other Term])) OR (sport*[Text Word])** 9. **((recover*[Title/Abstract]) OR (recover*[Other Term])) OR (recover*[Text Word])** 10. **((fatigu*[Title/Abstract]) OR (fatigu*[Other Term])) OR (fatigu*[Text Word])** 11. **((pre-perform*[Title/Abstract]) OR (pre-perform*[Other Term])) OR (pre-perform*[Text Word])** 12. **((compet*[Title/Abstract]) OR (compet*[Other Term])) OR (compet*[Text Word])** 13. **((train*[Title/Abstract]) OR (train*[Other Term])) OR (train*[Text Word])** 14. **((athlet*[Title/Abstract]) OR (athlet*[Other Term])) OR (athlet*[Text Word])** 15. **((coach*[Title/Abstract]) OR (coach*[Other Term])) OR (coach*[Text Word])** 16. **#2 OR #3 OR #4 OR #5 OR #6 OR #7** 17. **#8 OR #9 OR #10 OR #11 OR #12 OR #13 OR #14 OR #15** 18. #1 AND #16 AND #17 |
| **Additional hits** | 13 |

| **Total additional hits** | 84 |
| --- | --- |
| **After deduplication** | 32 |
| **Conflicts after abstract and title screening** | 0 |

**Manual Searches of Sport Psychology Journals (N = 7) Search (05.11.21)**

| Journal | Psychology of Sport and Exercise |
| --- | --- |
| Date | 05.11.21 |
| Search term | Taper |
| Limits | None |
| Additional hits | 1 |

| Journal | Journal of Sport and Exercise Psychology |
| --- | --- |
| Date | 05.11.21 |
| Search term | Taper |
| Limits | None |
| Additional hits | 0 |

| Journal | Journal of Applied Sport Psychology |
| --- | --- |
| Date | 05.11.21 |
| Search term | Taper |
| Limits | None |
| Additional hits | 1 |

| Journal | International Journal of Sport and Exercise Psychology |
| --- | --- |
| Date | 05.11.21 |
| Search term | Taper |
| Limits | None |
| Additional hits | 0 |

| Journal | Journal of Clinical Sport Psychology |
| --- | --- |
| Date | 05.11.21 |
| Search term | Taper |
| Limits | None |
| Additional hits | 0 |

| Journal | Journal of Sport Psychology in Action |
| --- | --- |
| Date | 05.11.21 |
| Search term | Taper |
| Limits | None |
| Additional hits | 0 |

| Journal | Sport, Exercise, and Performance Psychology |
| --- | --- |
| Date | 05.11.21 |
| Search term | Taper |
| Limits | None |
| Additional hits | 0 |
| **Total additional hits** | 2 |
| **After deduplication** | 2 |

| **Total hits after both searches** | 45 |
| --- | --- |
| **After both deduplications** | 40 |

| **Total hits after both searches** | 1708 |
| --- | --- |
| **Total after deduplication** | 1019 |
| **Total conflicts after abstract and title screening** | 52 (5.27%) |
